# Integrated Transcriptomic Analysis Identifies a Candidate INHBA/Activin A-Associated Macrophage–MuSC Communication Axis in Human Skeletal Muscle Ageing

**DOI:** 10.3390/ijms27188283

**Published:** 2026-09-17

**Authors:** Xuanchen Liu, Zehui Li, Yutong Yuan, Nuo Si, Ningbei Yin

**Affiliations:** Plastic Surgery Hospital, Chinese Academy of Medical Sciences and Peking Union Medical College, Beijing 100043, China

**Keywords:** skeletal muscle ageing, muscle stem cells, macrophages, single-cell RNA sequencing, cell–cell communication, INHBA/Activin A signalling

## Abstract

Skeletal muscle ageing is accompanied by impaired regeneration and remodelling of the multicellular niche, yet how age-related immune changes relate to altered muscle stem cell (MuSC) states in humans remains unclear. Here, we integrated bulk RNA-sequencing, scRNA-seq and an independent older adult cohort analysis to characterize age-associated changes in human skeletal muscle and identify candidate macrophage–MuSC signalling interactions. Bulk transcriptomic analysis revealed inflammatory activation, reduced mitochondrial and metabolic programmes, and increased macrophage-associated signatures in aged muscle. Single-cell analysis resolved distinct myeloid and MuSC states. Among aged myeloid populations, inflammatory macrophages exhibited the highest SenMayo score, whereas donor-level pseudobulk analysis identified age-related early-primed MuSCs (epMuSC) remodelling involving inflammatory, interferon-related, and extracellular matrix-associated programmes. Ligand–target modelling prioritized inhibin subunit beta A (INHBA)/Activin A as a candidate signal linking inflammatory macrophages to the aged epMuSC programme. This prediction was further supported by an independent older adult human muscle scRNA-seq cohort showing concordant INHBA expression in inflammatory macrophages and Activin receptor expression in epMuSCs. In primary human MuSCs, Activin A induced Activin-responsive genes and suppressed myogenic regulators, and these effects were attenuated by SB431542, a small-molecule inhibitor of ALK4/5/7 signalling. Together, these findings support a candidate INHBA-associated, cell state-resolved communication framework between inflammatory macrophages and epMuSC remodelling in aged human muscle, providing a potential link between immune remodelling and altered regenerative cell states.

## 1. Introduction

Skeletal muscle ageing is accompanied by progressive losses in muscle mass, strength, and regenerative capacity, which contribute to frailty, impaired mobility, and delayed recovery from injury [1]. These functional declines occur alongside mitochondrial dysfunction, chronic inflammation, extracellular matrix remodelling, and defective tissue repair [1]. Such changes involve not only myofibres but also the immune, stromal, vascular, and regenerative cells that form the muscle niche [2,3]. Age-related muscle dysfunction may therefore reflect disrupted coordination among niche populations as well as cell-intrinsic deterioration.

Muscle stem cells (MuSCs), also known as satellite cells, sustain muscle homeostasis and regenerate damaged fibres [4]. Ageing impairs MuSC activation, expansion, and differentiation through both cell-intrinsic changes and altered niche-derived signals [3,5,6]. MuSCs also occupy distinct states across quiescence, activation, and differentiation, with different self-renewal and lineage capacities [7,8]. Human single-cell studies have identified age-related changes in MuSC abundance and transcriptional heterogeneity [3,9]. However, which MuSC states are most vulnerable and which niche signals accompany their alteration remain unclear.

Macrophages regulate muscle repair through coordinated changes in their functional states, providing signals that control debris clearance, MuSC activation, and myogenic progression [10,11]. Ageing alters the composition and transcriptional profiles of resident and infiltrating myeloid populations, potentially disrupting these temporally regulated interactions [12,13]. However, the paracrine signals linking altered macrophage states to specific MuSC populations in ageing human muscle remain poorly defined. Inhibin subunit beta A (INHBA) encodes the inhibin βA subunit, and two βA subunits assemble to form the homodimeric ligand Activin A. It signals through ACVR2A or ACVR2B together with ACVR1B, leading to SMAD2/3-dependent transcription. Previous studies have established roles for Activin signalling in experimental muscle regeneration, myogenic differentiation, and muscle wasting [14,15]. These studies have primarily examined experimental injury, exogenous ligand exposure, or systemic pathway manipulation. The local cellular source of Activin-related signalling in ageing human muscle and its relationship to specific MuSC states therefore remain unclear.

Here, we sought to define how ageing-associated immune remodelling influences regenerative cell states in human skeletal muscle. By integrating bulk RNA-seq, single-cell RNA-seq, donor-level pseudobulk analysis, ligand–target modelling, and independent older adult cohort analysis, we identified a candidate relationship between INHBA-expressing inflammatory macrophages and early-primed MuSCs (epMuSCs), which represent a transcriptionally defined transitional state between quiescence and differentiation. We further examined INHBA localization in aged human muscle and evaluated primary human MuSC responses to Activin A and SB431542. Together, these analyses support a candidate cell state-resolved macrophage-to-epMuSC signalling framework in which INHBA/Activin A-associated Activin signalling may contribute to regenerative niche remodelling during human skeletal muscle ageing.

## 2. Results

### 2.1. Ageing Is Associated with Coordinated Inflammatory and Metabolic Transcriptomic Remodelling in Human Skeletal Muscle

**Figure 1:**
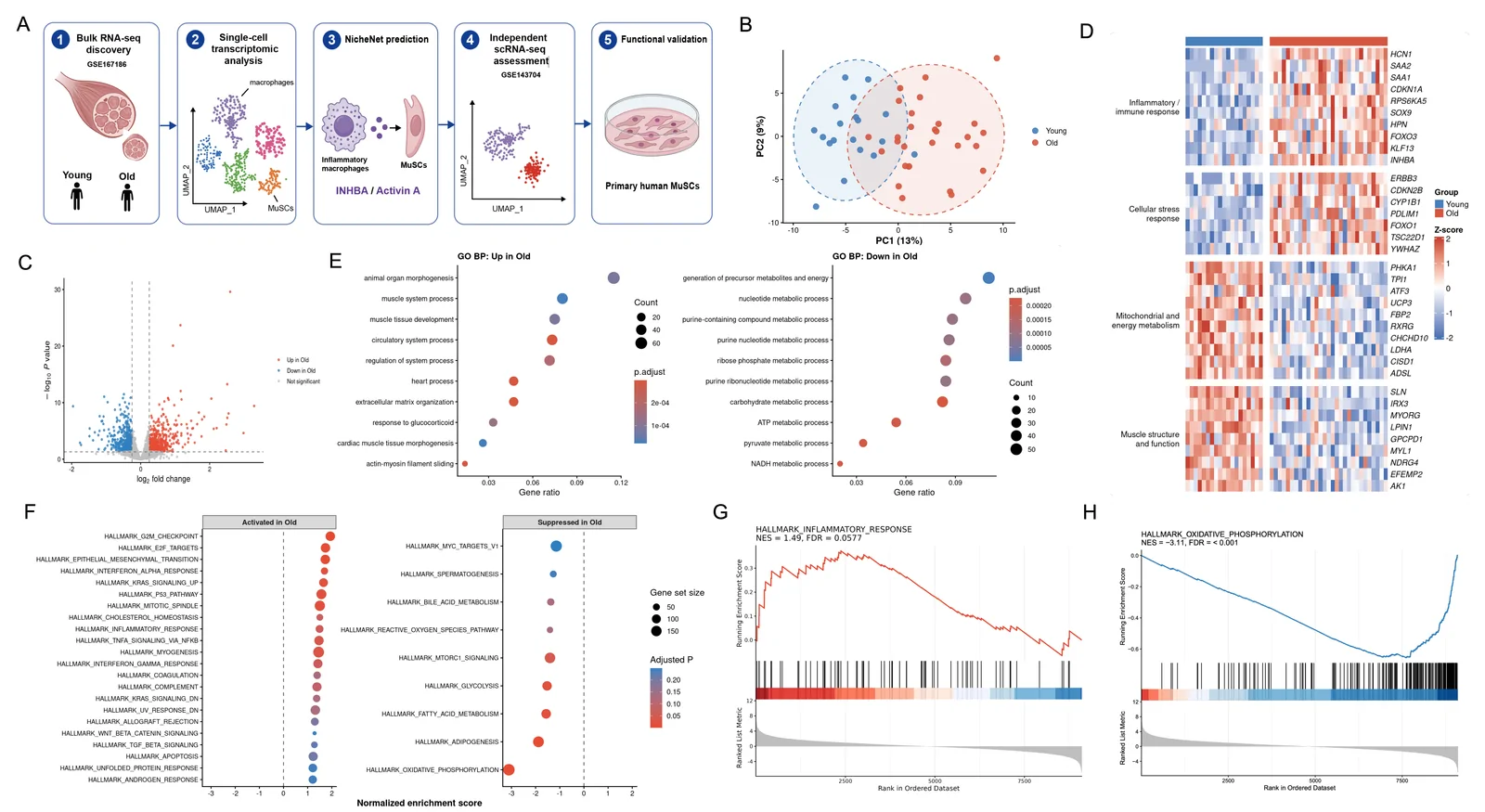
Age-associated inflammatory activation and metabolic decline in human skeletal muscle transcriptomes. (**A**) Overview of the integrated analytical workflow. (**B**) Principal component analysis (PCA) of bulk RNA-sequencing profiles from young and old human skeletal muscle samples. Dashed ellipses depict 95% data ellipses for each age group. (**C**) Volcano plot showing differentially expressed genes between young and old skeletal muscle. Red and blue dots indicate genes upregulated and downregulated in old skeletal muscle, respectively, whereas grey dots represent genes without significant changes. The horizontal dashed line indicates *p* = 0.05, and the vertical dashed lines indicate log_2_ fold changes of −0.25 and 0.25. (**D**) Heatmap showing representative ageing-associated genes grouped into functional categories, including inflammatory/immune response, cellular stress, mitochondrial metabolism and muscle-related functions. (**E**) Gene Ontology biological process enrichment analysis of genes upregulated and downregulated in old skeletal muscle. Dot size indicates gene count, and dot colour represents adjusted *p* value. (**F**) Hallmark gene set enrichment analysis summarizing age-associated pathway alterations. Normalized enrichment scores (NESs) indicate the direction and magnitude of pathway enrichment in old skeletal muscle. (**G**,**H**) Representative GSEA enrichment plots showing positively enriched inflammatory response (**G**) and negatively enriched oxidative phosphorylation (**H**) pathways in old skeletal muscle. Differentially expressed genes were identified using the criteria of *p* < 0.05 and |log2 fold change| ≥ 0.25. Gene set enrichment analyses were performed using MSigDB hallmark gene sets with false discovery rate (FDR) correction. The red curve in (**G**) and the blue curve in (**H**) indicate positive and negative enrichment in old skeletal muscle, respectively.

We first characterized age-associated transcriptional alterations in human skeletal muscle using bulk RNA-sequencing data from young and older individuals (Figure 1A). Principal component analysis revealed an age-related shift in the global transcriptional landscape (Figure 1B). Sample-to-sample correlation analysis showed overall consistency among the transcriptomic profiles (Figure S1A). Differential expression analysis identified widespread age-associated transcriptional changes (Figure 1C; Table S4). A heatmap of representative differentially expressed genes further demonstrated distinct expression patterns between young and aged skeletal muscle, with the selected genes grouped into functional categories related to inflammatory and immune responses, cellular stress, mitochondrial and energy metabolism, and muscle structure and function (Figure 1D).

Functional enrichment analysis revealed coordinated biological remodelling during skeletal muscle ageing. Genes upregulated in aged skeletal muscle were mainly enriched in processes related to muscle organization, extracellular matrix remodelling, and cytoskeletal functions, whereas downregulated genes were predominantly associated with metabolic processes (Figure 1E). Hallmark gene set enrichment analysis further demonstrated increased activity of inflammatory and stress-related programmes together with reduced activity of mitochondrial and metabolism-associated pathways in aged skeletal muscle (Figure 1F). Representative GSEA plots showed a positive near-threshold enrichment trend for inflammatory response and negative enrichment of oxidative phosphorylation in aged muscle (Figure 1G,H). KEGG enrichment, additional hallmark GSEA plots, and GSVA analyses further supported these coordinated pathway-level alterations (Figure S1B–H). Collectively, these results indicate that aged human skeletal muscle is characterized by enhanced inflammatory programmes and reduced mitochondrial and metabolic activity.

### 2.2. Ageing Remodels the Immune Microenvironment of Human Skeletal Muscle with Increased Macrophage-Associated Signatures

**Figure 2:**
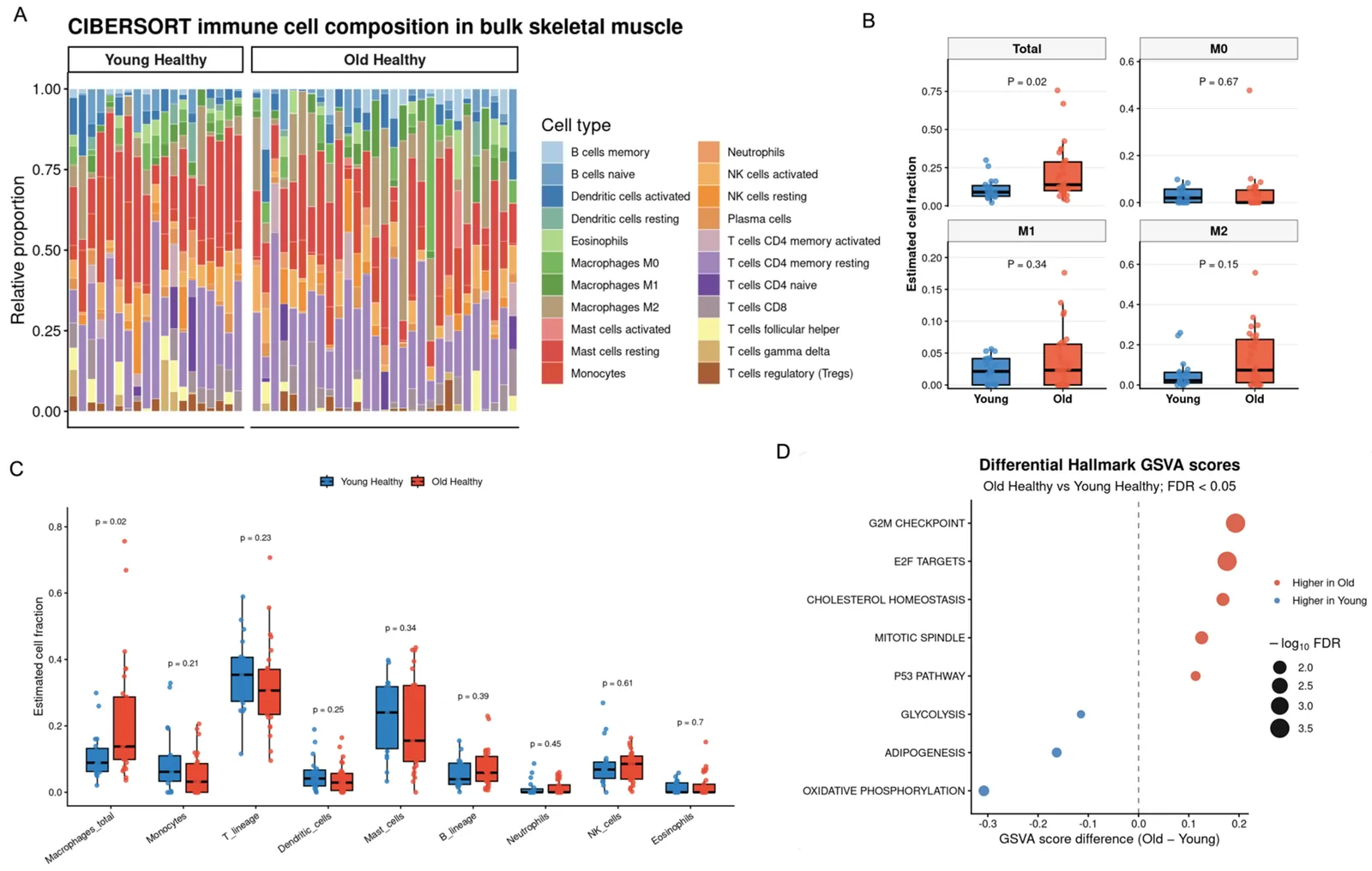
Age-related remodelling of the immune microenvironment in skeletal muscle. (**A**) Relative abundance of immune cell populations estimated from bulk skeletal muscle transcriptomes using CIBERSORT. Each stacked bar represents one sample, grouped by age. (**B**) Comparison of CIBERSORT-derived macrophage-related fractions, including total macrophages and the M0, M1, and M2 macrophage subsets, between young and old skeletal muscle. (**C**) Estimated abundance of selected immune cell populations in young and old skeletal muscle. Each point represents one sample, and box plots indicate the median and interquartile range. (**D**) Differential hallmark pathway activity between old and young skeletal muscle, assessed by GSVA followed by limma analysis. Positive values indicate higher pathway activity in old skeletal muscle, whereas negative values indicate higher activity in young skeletal muscle. *p* values were adjusted using the Benjamini–Hochberg method.

Because bulk tissue transcriptomes reflect both cellular composition and cell-intrinsic transcriptional changes, we used CIBERSORT to estimate immune cell abundance. Immune cell composition varied across young and old skeletal muscle samples (Figure 2A). The estimated total macrophage fraction was significantly higher in old skeletal muscle (*p* = 0.02), whereas the CIBERSORT-defined M0, M1, and M2 macrophage subsets did not differ significantly between groups (Figure 2B). No significant age-associated differences were detected in the other immune cell populations examined (Figure 2C).

Differential hallmark GSVA analysis showed increased activity of cell cycle- and stress-associated programmes, including the G2M checkpoint, E2F targets, mitotic spindle, and p53 pathway, together with reduced glycolysis, adipogenesis, and oxidative phosphorylation in old skeletal muscle (FDR < 0.05; Figure 2D). These findings suggest that macrophages represent a prominent immune component associated with ageing-related skeletal muscle alterations and therefore motivated subsequent single-cell analysis of macrophage states.

### 2.3. Single-Cell Transcriptomic Profiling Defines Cellular Heterogeneity and Ageing-Associated Alterations in Human Skeletal Muscle

**Figure 3:**
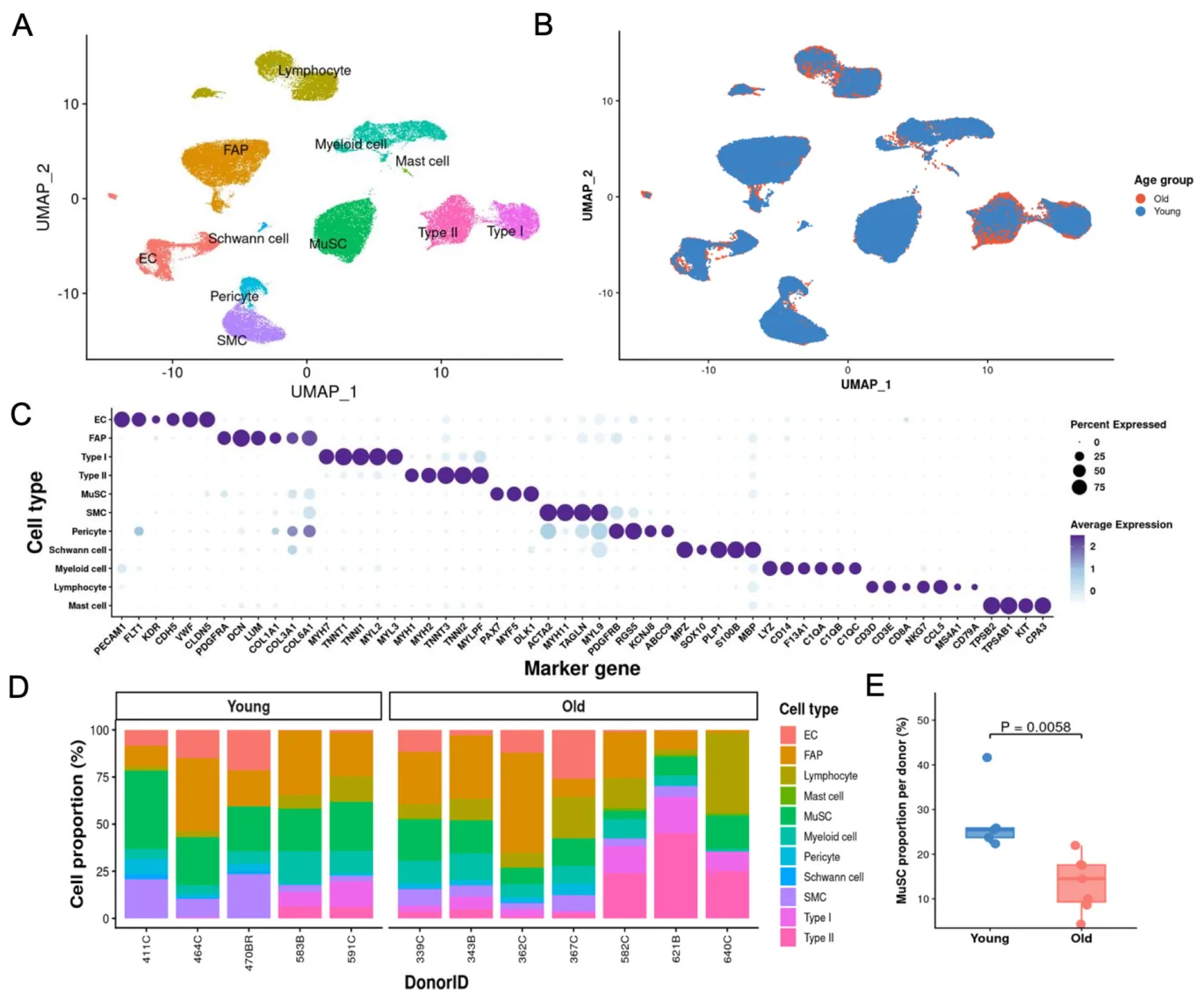
Single-cell transcriptomic profiling defines cellular composition and ageing-associated alterations in human skeletal muscle. (**A**) Uniform manifold approximation and projection (UMAP) visualization of human skeletal-muscle scRNA-seq data, coloured by annotated cell type. Major populations include fibro-adipogenic progenitors (FAPs), endothelial cells (ECs), myeloid cells, lymphocytes, mast cells, pericytes, Schwann cells, smooth muscle cells (SMCs), muscle stem cells (MuSCs), and type I and type II myofibre-derived fragments. (**B**) UMAP visualization coloured by age group, showing the distribution of cells from young and old donors. (**C**) Dot plot showing canonical marker gene expression used for cell-type annotation. Dot size indicates the percentage of cells expressing each gene, and colour indicates the average scaled expression level. (**D**) Cell-type composition at the donor level, stratified by age group. Each bar represents one donor. (**E**) Comparison of MuSC proportions between young and old donors. Each point represents one donor, and box plots indicate the median and interquartile range.

To define age-associated cellular alterations at single-cell resolution, we analysed scRNA-seq from young and old human skeletal muscle. The single-cell component initially comprised 90,902 cells, including 41,261 cells from young donors and 49,641 cells from old donors. Following quality control and integration, unsupervised clustering identified the major skeletal muscle cell populations, including fibro-adipogenic progenitors (FAPs), endothelial cells, myeloid cells, lymphocytes, mast cells, pericytes, Schwann cells, smooth muscle cells, muscle stem cells (MuSCs), and type I and type II myofibre-derived fragments (Figure 3A). Cells from both age groups were distributed across the major populations, and cell identities were confirmed by canonical lineage markers (Figure 3B,C).

Donor-level analysis revealed age-associated differences in cellular composition across skeletal muscle populations (Figure 3D). Notably, the proportion of MuSCs was significantly reduced in old donors compared with young donors (*p* = 0.0058; Figure 3E). Quality control assessment and dataset integration are shown in Figure S2, including distributions of detected genes, UMI counts, mitochondrial transcript fraction, donor-level cell numbers, principal component selection, and donor mixing after integration (Figure S2A–E). These analyses provided a cellular reference framework for subsequent characterization of age-associated transcriptional changes and intercellular signalling.

### 2.4. Inflammatory Macrophages Display a Senescence-Associated Transcriptional State, Whereas epMuSCs Undergo Age-Related Transcriptional Remodelling

**Figure 4:**
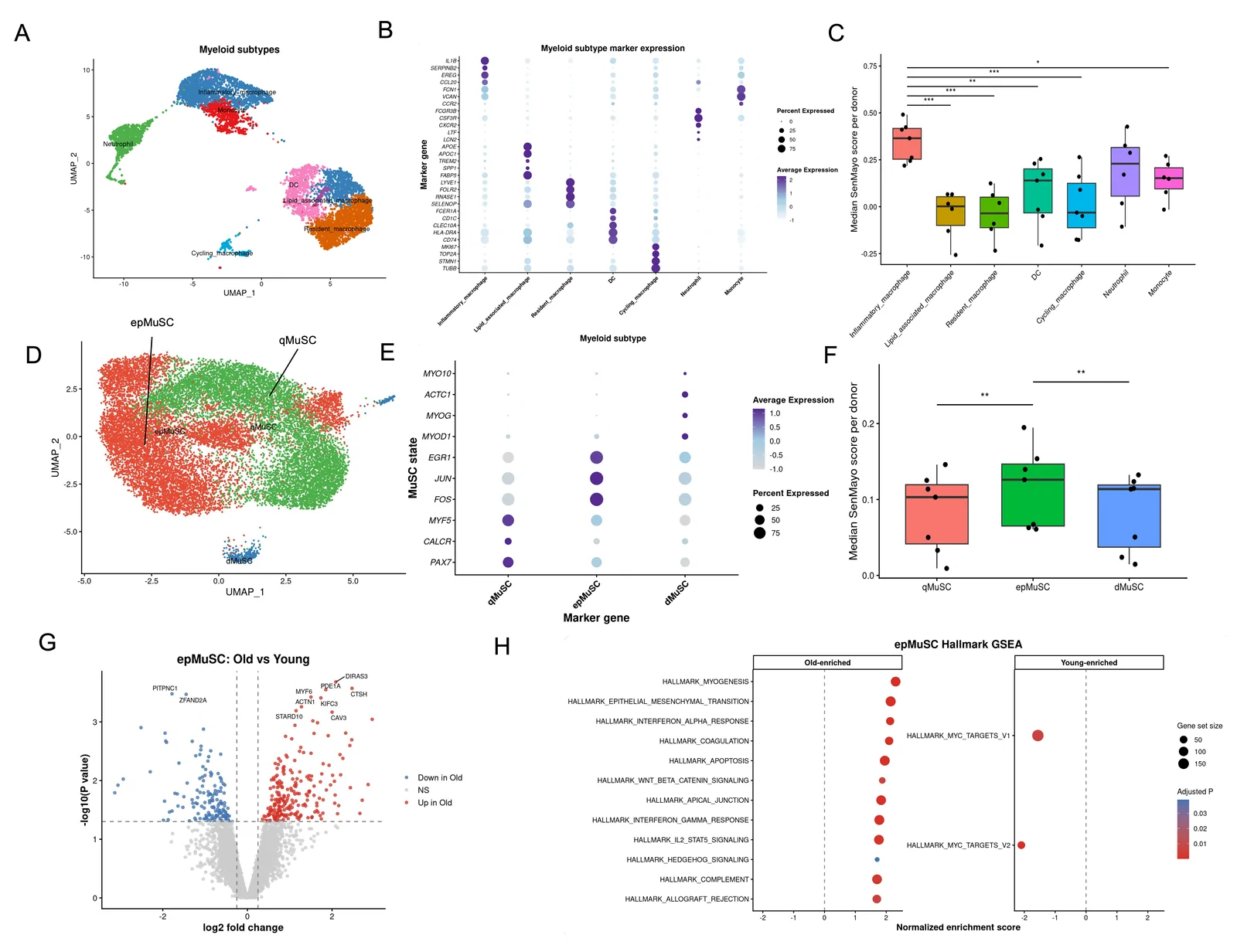
Ageing-associated transcriptional remodelling of myeloid cells and muscle stem cells. (**A**) Uniform manifold approximation and projection (UMAP) of the myeloid cell compartment, coloured by annotated myeloid subtype. (**B**) Dot plot of canonical marker gene expression used for myeloid subtype annotation. Dot size indicates the percentage of cells expressing each gene, and colour indicates the average scaled expression. (**C**) Donor-level SenMayo scores across myeloid subtypes in aged samples. Each point represents one donor, and box plots indicate the median and interquartile range. Statistical comparisons were performed using donor-aware linear mixed-effects models with Benjamini–Hochberg-adjusted post hoc contrasts. * Adjusted *p* < 0.05, ** adjusted *p* < 0.01, and *** adjusted *p* < 0.001. (**D**) UMAP of muscle stem cells (MuSCs), coloured by transcriptional state. (**E**) Dot plot of marker gene expression defining the MuSC states. Dot size indicates the percentage of cells expressing each gene, and colour indicates the average scaled expression. (**F**) Comparison of donor-level SenMayo scores across MuSC states. Statistical differences were assessed using linear mixed-effects models with MuSC state as a fixed effect and donor identity as a random effect. ** Adjusted *p* < 0.01. (**G**) Volcano plot showing genes with nominal age-associated expression changes between old and young epMuSCs. Red and blue dots indicate genes meeting nominal significance criteria (*p* < 0.05) with increased or decreased expression in old epMuSCs, respectively, whereas grey dots indicate genes not meeting nominal significance criteria. The horizontal dashed line indicates *p* = 0.05, and the vertical dashed lines indicate log_2_ fold changes of −0.25 and 0.25. (**H**) Hallmark gene set enrichment analysis (GSEA) of age-associated transcriptional changes in epMuSCs. Dot position indicates the normalized enrichment score, dot size indicates gene set size, and dot colour represents the adjusted *p* value.

Given the immune alterations identified in bulk transcriptomic analyses, we further resolved the myeloid compartment into distinct subpopulations, including inflammatory macrophages, lipid-associated macrophages, resident-like macrophages, dendritic cells, monocytes, neutrophils, and cycling macrophages (Figure 4A). Canonical marker expression supported these annotations (Figure 4B).

Donor-level SenMayo scores varied across myeloid subtypes in aged skeletal muscle, with inflammatory macrophages showing the highest median score (Figure 4C). Linear mixed-effects modelling accounting for donor identity confirmed significant differences among myeloid states, with inflammatory macrophages exhibiting higher SenMayo scores than several other myeloid populations.

Within the MuSC compartment, unsupervised clustering identified quiescent MuSCs, early-primed MuSCs, and differentiating MuSCs (Figure 4D), which were supported by state-specific marker expression (Figure 4E). SenMayo scores differed significantly across the three MuSC states, with epMuSCs exhibiting the highest median score among the identified MuSC populations. Pairwise comparisons further showed that epMuSCs had higher SenMayo scores than other MuSC states after donor-aware mixed-effects modelling and multiple testing correction (Figure 4F). Pairwise contrasts were adjusted using the Benjamini–Hochberg method.

Notably, donor-level pseudobulk analysis identified nominally altered candidate genes in epMuSCs (Figure 4G). Ranked gene set enrichment analysis further revealed age-associated remodelling of inflammatory, interferon-related, and extracellular matrix-associated transcriptional programmes (Figure 4H). Collectively, epMuSCs exhibited age-associated transcriptional programme remodelling, characterized primarily by pathway-level changes involving inflammatory, interferon-related, and extracellular matrix-associated programmes.

### 2.5. Ageing-Associated Inflammatory Macrophages Exhibit an INHBA-Enriched Signalling Programme Linked to epMuSC Remodelling

**Figure 5:**
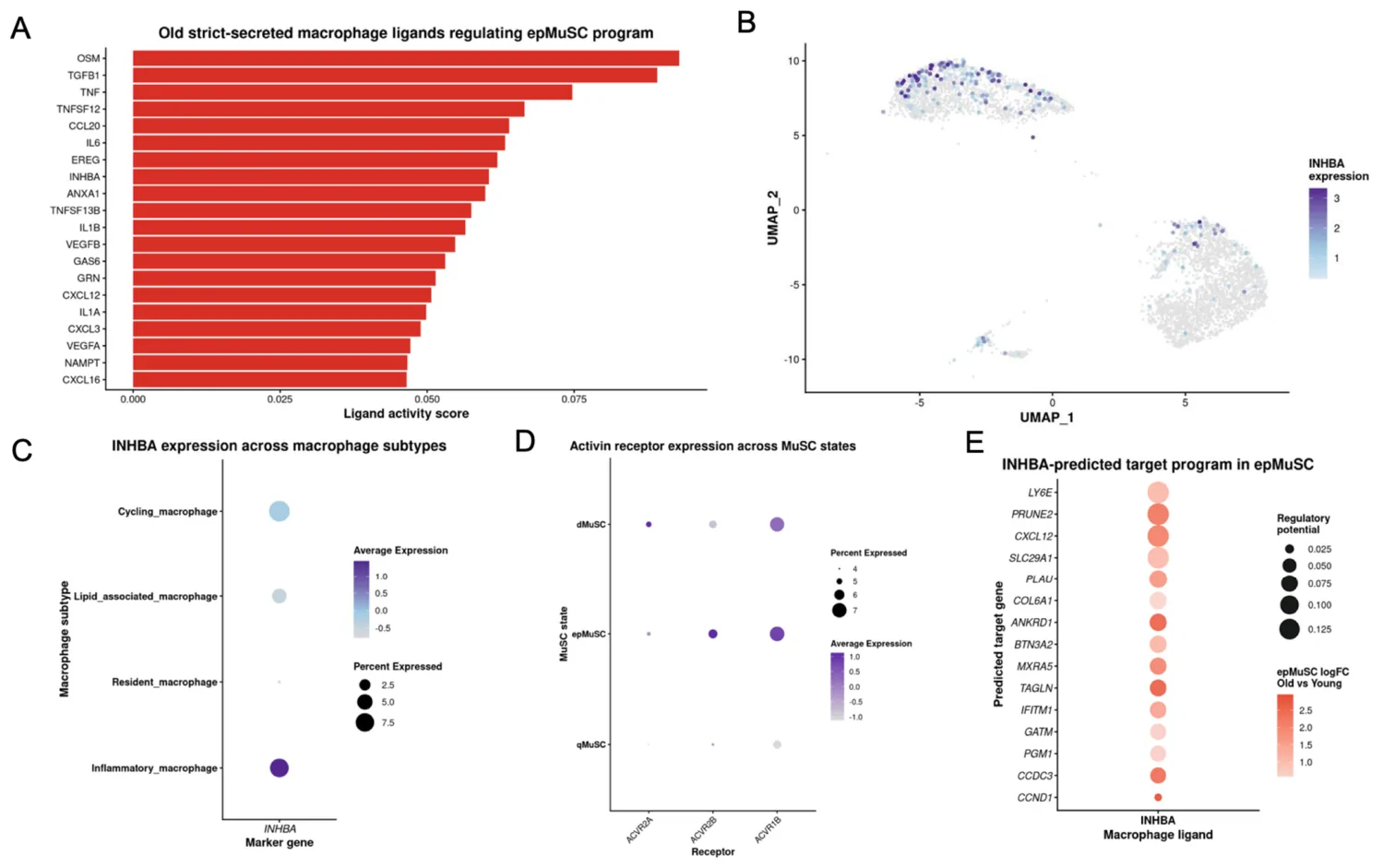
Predicted INHBA-associated macrophage-to-MuSC signalling in aged skeletal muscle. (**A**) Ranked macrophage-derived ligands predicted to regulate the epMuSC transcriptional programme in aged skeletal muscle. Ligands are ordered by ligand activity score. (**B**) Feature plot showing INHBA expression across macrophage cells in the UMAP space. (**C**) Dot plot showing INHBA expression across macrophage subtypes. Dot size indicates the percentage of cells expressing INHBA, and dot colour indicates the average scaled expression. (**D**) Dot plot showing expression of activin receptors across MuSC states. Dot size indicates the percentage of cells expressing each receptor, and dot colour indicates the average scaled expression. (**E**) Predicted INHBA target genes in epMuSCs. Dot size indicates regulatory potential, and dot colour indicates the log2 fold change in epMuSCs from old versus young donors.

To identify candidate signals linking inflammatory macrophages to the age-associated transcriptional programme of epMuSCs, we performed NicheNet ligand–target analysis using inflammatory macrophages and epMuSCs from old donors as the sender and receiver populations, respectively. Among the macrophage-derived ligands predicted to regulate genes upregulated in old epMuSCs, INHBA was prioritized among macrophage-derived candidate ligands (Figure 5A).

INHBA was preferentially expressed in inflammatory macrophages among the analysed macrophage states (Figure 5B,C). In parallel, epMuSCs expressed the Activin receptor components ACVR2A, ACVR2B, and ACVR1B (Figure 5D). The predicted INHBA-associated target programme included multiple genes upregulated in old epMuSCs, several of which showed relatively high regulatory potential scores (Figure 5E). Together, these findings support a candidate INHBA/Activin A-associated macrophage-to-epMuSC signalling framework linked to the aged epMuSC transcriptional programme.

### 2.6. Independent Older Adult Cohort Support for the INHBA-Associated Macrophage–epMuSC Expression Relationship

**Figure 6:**
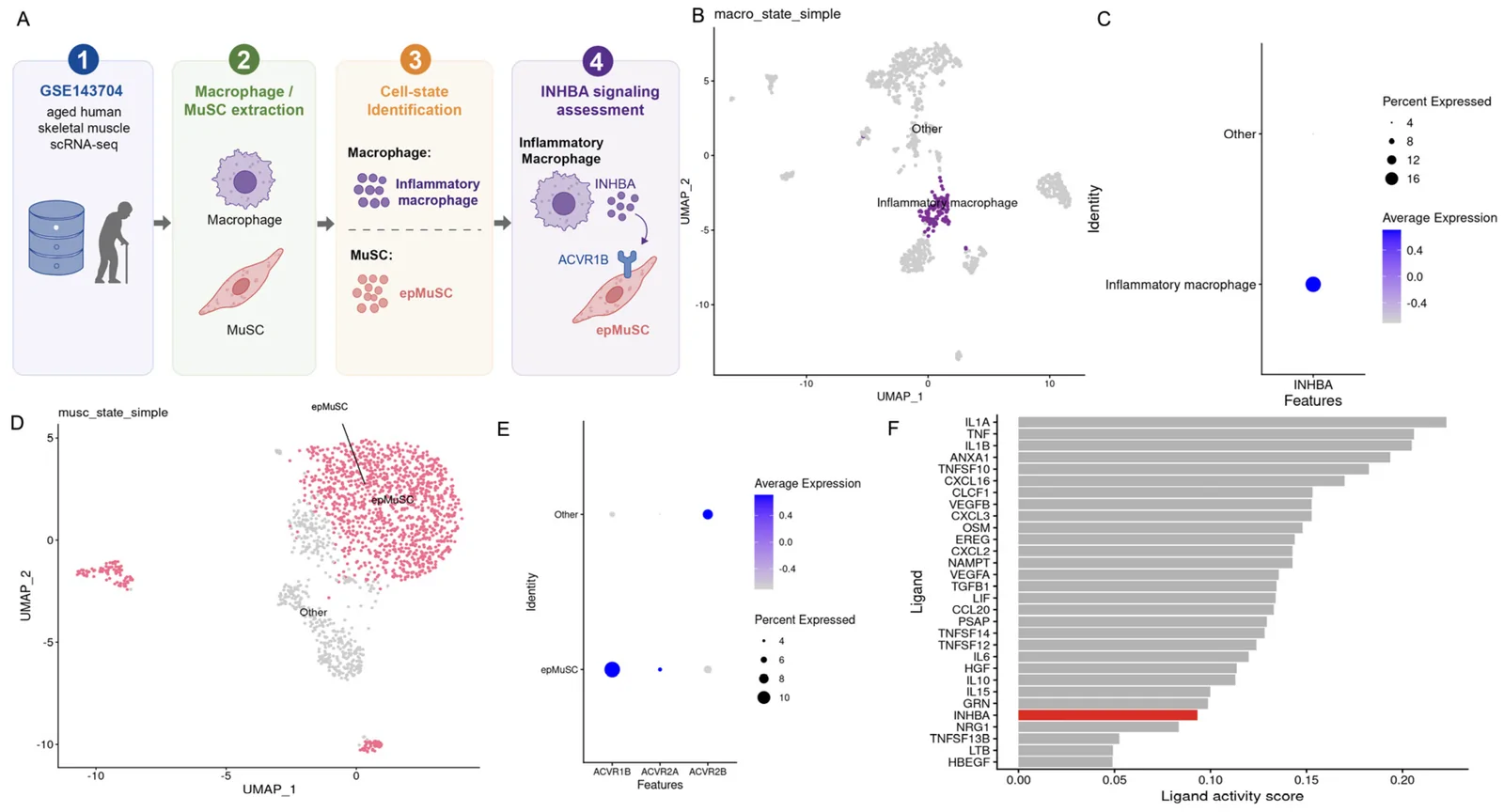
Independent older adult cohort support for the INHBA-associated macrophage–epMuSC signalling framework in human skeletal muscle. (**A**) Overview of the independent older adult cohort analysis using the GSE143704 human skeletal muscle scRNA-seq dataset. (**B**) UMAP visualization of macrophage populations in GSE143704. Inflammatory macrophages were highlighted, whereas other macrophage populations were grouped as “Other” for focused INHBA expression analysis. (**C**) Dot plot showing INHBA expression across inflammatory macrophages and other macrophage populations. Dot size represents the percentage of cells expressing INHBA, and colour indicates the average expression level. (**D**) UMAP visualization of MuSC populations. epMuSCs were highlighted and compared with other MuSC populations grouped as “Other”. (**E**) Dot plot showing the expression of Activin receptor components, including ACVR1B, ACVR2A, and ACVR2B, across epMuSCs and other MuSC populations. Dot size represents the percentage of cells expressing each gene, and colour indicates the average expression level. (**F**) NicheNet ligand activity ranking in the independent older adult cohort using inflammatory macrophages as sender cells and epMuSCs as receiver cells. INHBA was highlighted among candidate macrophage-derived ligands associated with the epMuSC transcriptional program. The straight arrows indicate the analytical workflow, and the curved arrow indicates the predicted direction of signalling from inflammatory macrophages to epMuSCs.

To obtain independent older adult cohort support for the NicheNet-predicted INHBA-associated macrophage–epMuSC signalling framework, we analysed an external human skeletal muscle scRNA-seq dataset, comprising 18,320 cells from nine donors aged 54–81 years (GSE143704) (Figure 6A). Macrophage and MuSC populations were extracted and re-clustered to resolve relevant cellular states.

Within the myeloid compartment, inflammatory macrophages were identified as a distinct population, whereas other macrophage states were grouped as “Other” for focused INHBA expression analysis (Figure 6B). INHBA expression was predominantly detected in inflammatory macrophages compared with other macrophage populations, supporting inflammatory macrophages as an INHBA-associated cellular source (Figure 6C).

In parallel, MuSC analysis identified an epMuSC population (Figure 6D). Expression analysis demonstrated the presence of Activin receptor components, including ACVR1B, within epMuSCs (Figure 6E).

Applying the NicheNet ligand–target framework using the predefined epMuSC-associated target programme, the older adult cohort retained INHBA among macrophage-derived candidate ligands associated with the epMuSC transcriptional programme (Figure 6F). Together, these analyses provided support for the presence of an INHBA-associated macrophage–epMuSC expression framework in an independent older adult human skeletal muscle cohort.

### 2.7. Functional Assessment of Activin A Pathway Responsiveness in Primary Human MuSCs

Based on the computational prediction, we next examined whether primary human MuSCs are responsive to Activin A stimulation. To provide supportive evidence for the cellular context of the predicted signalling framework, we first examined INHBA localization in human skeletal muscle tissue and assessed macrophage-associated INHBA expression and Activin A release, and then evaluated responses of primary human MuSCs to exogenous Activin A with or without SB431542.

**Figure 7:**
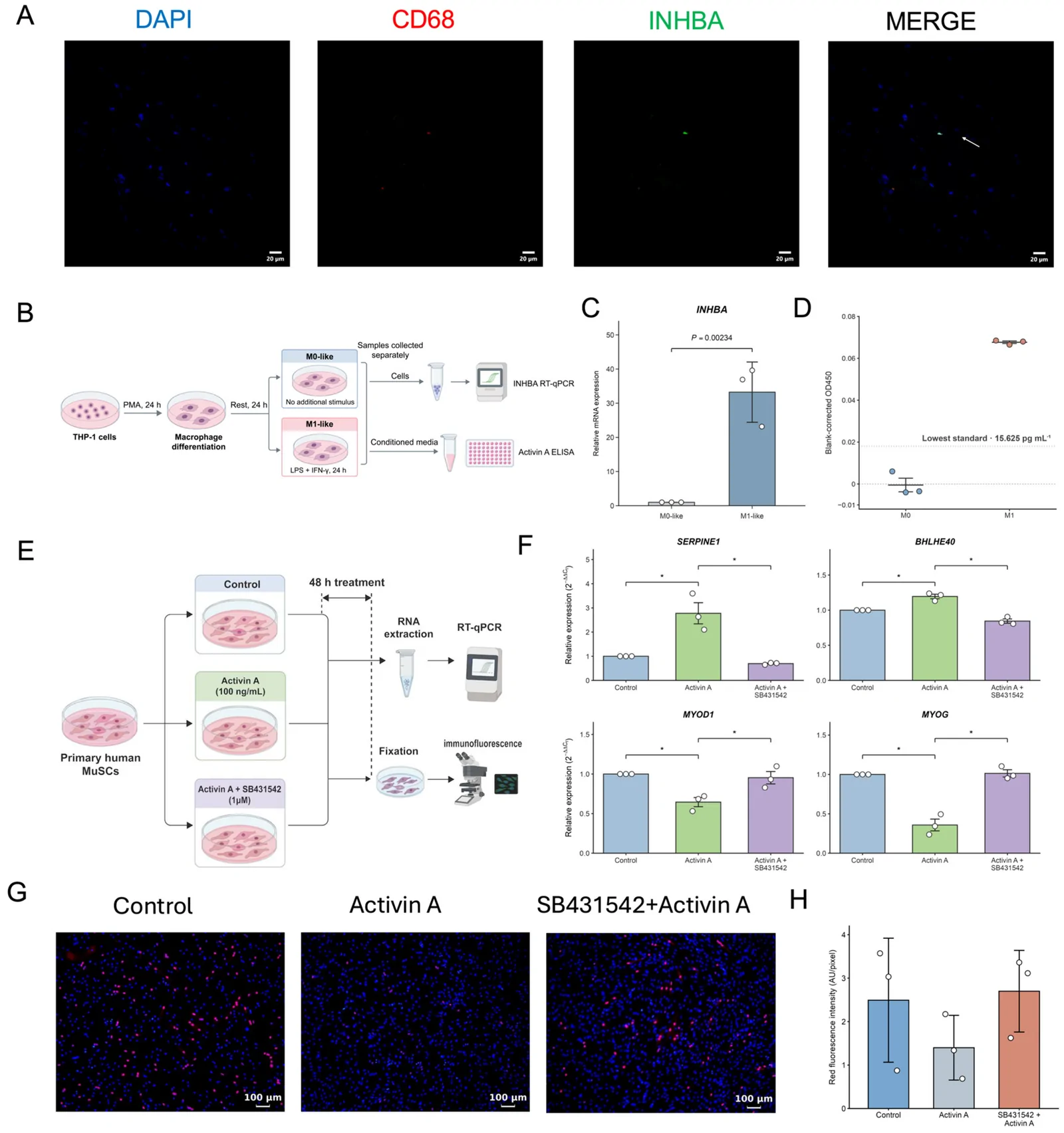
Macrophage-associated INHBA expression and Activin A responsiveness in primary human MuSCs. (**A**) Representative immunofluorescence images showing co-expression of CD68 (red) and INHBA (green) in human skeletal muscle tissue. Nuclei were counterstained with DAPI (blue). Arrows indicate representative CD68^+^INHBA^+^ cells. Scale bar, 20 μm. (**B**) Schematic illustration of THP-1-derived macrophage differentiation and polarization. Following PMA treatment and a resting period, macrophages were maintained without additional polarization stimuli (M0-like) or treated with LPS and IFN-γ for 24 h (M1-like). Cells and conditioned media were collected separately for INHBA RT–qPCR and Activin A ELISA, respectively. (**C**) RT–qPCR analysis of INHBA expression in THP-1-derived M0-like and M1-like macrophages. Expression is presented relative to the M0-like condition, with individual data points shown for three independent experiments per condition. The *p* value for the between-group comparison is indicated. (**D**) Activin A ELISA signals in conditioned media from M0-like and M1-like macrophages, expressed as blank-corrected absorbance at 450 nm (OD450). Each point represents the mean of technical duplicates for one sample, with three samples per condition. Horizontal lines and error bars indicate the mean ± SD. The dotted reference line above zero indicates the signal corresponding to the lowest non-zero standard. (**E**) Schematic illustration of the experimental design for Activin A stimulation and pharmacological pathway inhibition in primary human MuSCs. Cells were treated with vehicle control, Activin A (100 ng/mL), or Activin A combined with SB431542 (1 μM) for 48 h, followed by transcriptional and myogenic regulatory analyses. (**F**) RT–qPCR analysis of SERPINE1, BHLHE40, MYOD1, and MYOG expression in primary human MuSCs following treatment with vehicle control, Activin A, or Activin A plus SB431542. Bars represent the mean ± SEM, and each point represents one independent donor (*n* = 3). Two-sided paired *t*-tests were performed on donor-level ΔCt values for the two biologically defined comparisons per gene: Activin A versus control and Activin A plus SB431542 versus Activin A. The resulting eight *p* values were adjusted collectively using the Benjamini–Hochberg procedure. * Adjusted *p* < 0.05. (**G**) Representative immunofluorescence images of MYOG (red) and DAPI (blue) in primary human MuSCs following the indicated treatments. (**H**) Quantification of MYOG fluorescence intensity. Bars represent the mean ± SEM, and each point represents one independent donor (*n* = 3). Statistical comparisons were performed using donor-matched paired analyses with Benjamini–Hochberg correction.

Immunofluorescence staining identified representative CD68^+^INHBA^+^ cells, providing supportive evidence for macrophage-associated INHBA expression within human skeletal muscle tissue (Figure 7A).

To complement the tissue localization findings, we assessed INHBA mRNA expression in THP-1-derived M0-like and M1-like macrophages by RT-qPCR and Activin A in their conditioned media by ELISA (Figure 7B). INHBA mRNA expression was significantly higher in M1-like macrophages than in M0-like macrophages, with an approximately 30-fold increase (*p* < 0.01; Figure 7C). Consistent with this transcriptional difference, M1-like macrophage-conditioned medium showed higher blank-corrected ELISA signals than M0-like macrophage-conditioned medium, whereas M0-like signals remained close to assay backgrounds (Figure 7D). The estimated Activin A concentration in conditioned medium from M1-like macrophages was approximately 70.4 ± 1.4 pg/mL (mean ± SD; range, 68.8–71.5 pg/mL). Signals from M0-like samples were below the lowest non-zero standard and were therefore not converted to numerical concentrations.

Primary human MuSCs were subsequently exposed to Activin A, with or without SB431542, for 48 h (Figure 7E). Activin A increased the expression of the Activin-responsive genes SERPINE1 and BHLHE40 and reduced the expression of the myogenic regulators MYOD1 and MYOG relative to the donor-matched control condition. Co-treatment with SB431542 attenuated each of these Activin A-associated transcriptional changes. All eight donor-matched comparisons remained significant after Benjamini–Hochberg correction (adjusted *p* = 0.0471–0.0485; Figure 7F). These results support the involvement of SB431542-sensitive signalling in the transcriptional responses induced by Activin A.

Consistent with the RT-qPCR results, MYOG immunofluorescence showed a reduction following Activin A treatment and a recovery trend after SB431542 co-treatment, although these changes did not reach statistical significance after multiple comparison correction (Figure 7G,H). Together, these findings indicate that primary human MuSCs are responsive to Activin A stimulation, with associated changes in downstream transcriptional and myogenic regulatory programmes.

## 3. Discussion

**Figure 8:**
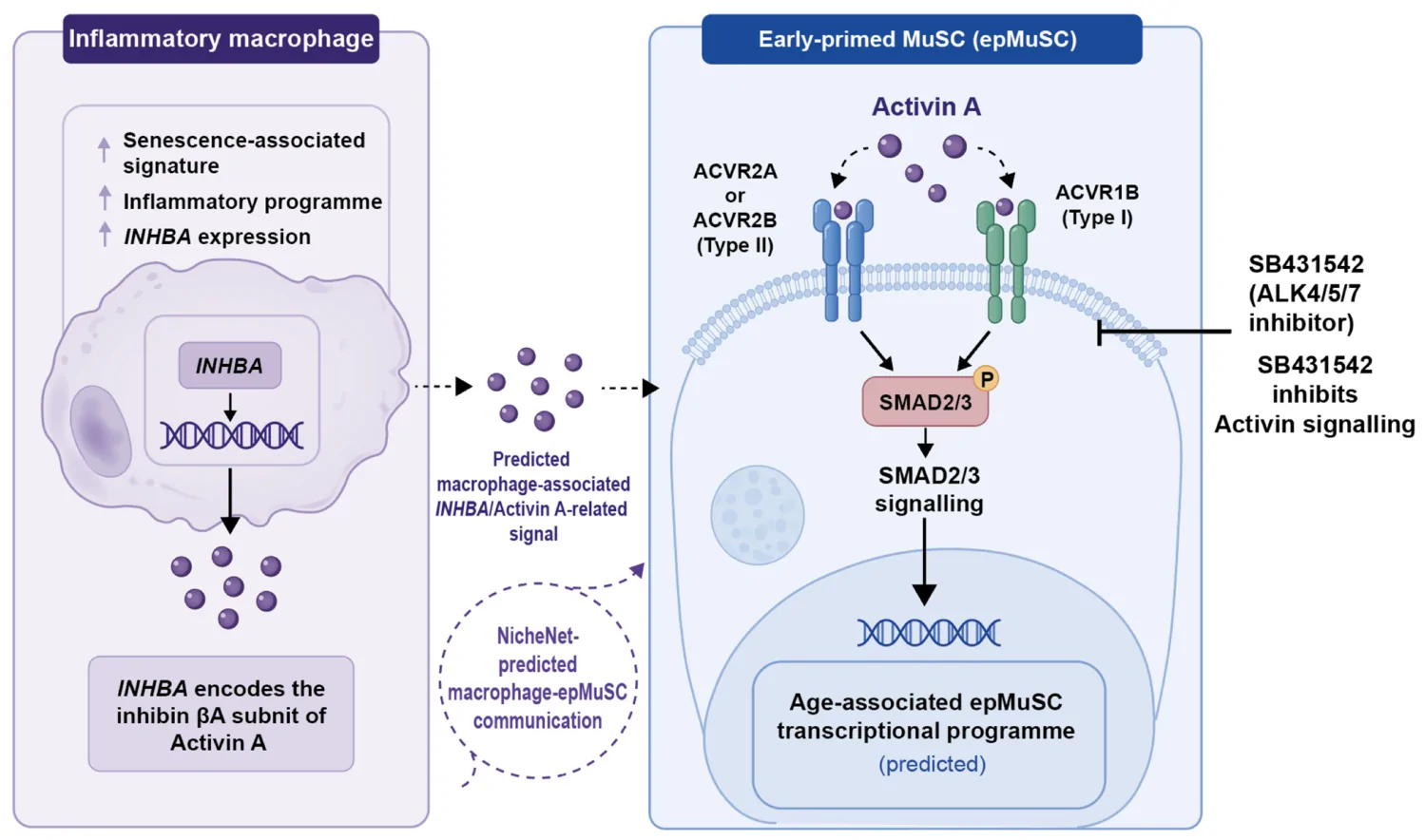
Model of ageing-associated macrophage–MuSC niche remodelling through INHBA/Activin A signalling. Schematic illustration showing the proposed interaction between inflammatory macrophages and epMuSCs during skeletal-muscle ageing. Inflammatory macrophages in aged skeletal muscle exhibit a prominent senescence-associated transcriptional state and express INHBA. INHBA encodes the inhibin βA subunit that contributes to the formation of Activin A, which is predicted to signal through ACVR2A/ACVR2B–ACVR1B receptors and engage SMAD2/3-associated programmes in epMuSCs. Pharmacological inhibition of ALK4/5/7 signalling by SB431542 attenuates Activin A responses. This model proposes a potential role for INHBA/Activin A signalling in regulating age-associated epMuSC states and myogenic regulatory responses.

Age-related regenerative decline has commonly been attributed to the deterioration of myofibres and loss of MuSC function [4,16]. However, MuSC behaviour is shaped by signals from a multicellular niche [17], and recent human cell atlases have revealed extensive ageing-associated changes across the immune and regenerative compartments of skeletal muscle [2,3]. Here, we identify an ageing-associated immune–regenerative niche remodelling process characterized by alterations in inflammatory macrophages and epMuSCs, with INHBA/Activin A emerging as a candidate signal linking these cell states (Figure 8). Primary human MuSC experiments further demonstrate responsiveness to Activin A. Together, these findings support a broader view of skeletal muscle ageing as a disorder of cellular coordination, in which altered communication between immune and regenerative states contributes to regenerative decline.

At the tissue level, aged skeletal muscle showed enrichment of inflammatory programmes and reduced representation of mitochondrial and metabolic programmes, consistent with previous transcriptomic analyses of healthy human ageing [18]. The coexistence of these changes suggests that inflammation and metabolic decline are not isolated features but components of a broadly altered tissue environment. Such an environment may expose regenerative cells to altered inflammatory and metabolic cues that influence activation and tissue repair, thereby providing a tissue-level explanation for the cellular changes resolved by single-cell analysis. The concurrent inflammatory and metabolic remodelling may also be considered in the context of the mechanistic target of rapamycin (mTOR) signalling, which connects nutrient sensing with cellular function and ageing [19,20,21].

Among the MuSC populations examined, epMuSCs showed pronounced age-associated transcriptional remodelling. Consistent with this transcriptional remodelling, epMuSCs also exhibited the highest SenMayo-associated programme among MuSC states. Previous studies have shown that the aged niche disrupts MuSC quiescence [6], while human single-cell analyses have distinguished quiescent from early-activated MuSC populations and reported an age-related loss of transcriptional heterogeneity [7,9]. In our analysis, aged epMuSCs were characterized by inflammatory, interferon-related, and extracellular matrix-associated programmes. The concurrence of these programmes suggests exposure to multiple niche-derived inputs rather than the disruption of a single pathway. Persistent inflammatory and interferon signalling may interfere with orderly myogenic progression, whereas extracellular matrix remodelling may alter the mechanical and molecular cues received by MuSCs. Because epMuSCs occupy a transition between quiescence and differentiation, these combined inputs may disrupt progression through early activation and contribute to an altered transitional state [8,16].

Macrophage function during muscle regeneration similarly depends on coordinated state transitions rather than fixed polarization identities [22]. Macrophage-derived signals provide transient support for MuSC activation and tissue repair [10,11,23], while ageing may disrupt these coordinated macrophage programmes in human muscle [17,24]. Experimental work has also linked reduced IFN-γ responsiveness in a macrophage subset to impaired satellite cell proliferation [13]. In our aged samples, inflammatory macrophages exhibited the highest SenMayo score among the identified myeloid populations. This score is best interpreted as an inflammatory and stress-associated programme with senescence-related features rather than definitive cellular senescence [25]. The coexistence of these features suggests that inflammatory macrophages may modify the regenerative niche through altered secretory outputs, providing the rationale for investigating their signals to epMuSCs.

Ligand–target analysis prioritized INHBA/Activin A as a possible link between inflammatory macrophages and the aged epMuSC programme. This prioritization was supported by INHBA expression in the sender population, expression of compatible Activin receptors in epMuSCs, and overlap between predicted targets and genes altered in aged epMuSCs. The additional THP-1-derived macrophage experiments showed higher *INHBA* expression and extracellular Activin A immunoreactivity under M1-like conditions, complementing the tissue localization findings and supporting macrophages as a potential source of Activin A. The independent older adult cohort further supported the presence of an INHBA-associated macrophage–epMuSC expression pattern across human muscle datasets. Receptor expression establishes that epMuSCs have the capacity to receive Activin-related signals, but it does not demonstrate selective responsiveness relative to other MuSC states. Activin signalling has established roles in myoblast differentiation, muscle wasting, and regenerative regulation: Activin A can inhibit myoblast differentiation, increased Activin levels promote muscle wasting, and manipulation of Activin type II receptor signalling influences regenerative capacity [14,15,26]. These studies have largely relied on cultured myoblasts, experimental injury, or systemic pathway manipulation and have not defined a local macrophage source together with a transitional MuSC recipient in ageing human muscle. The contribution of this study lies in defining a candidate cellular context for Activin-related signalling in ageing human muscle by linking INHBA-expressing inflammatory macrophages with epMuSCs as a potential responding state.

Functional experiments demonstrated that primary human MuSCs are responsive to Activin A stimulation. Activin A increased SERPINE1 and BHLHE40 expression, reduced MYOD1 and MYOG expression, and decreased MYOG immunofluorescence, consistent with previous evidence that Activin signalling suppresses human myoblast differentiation [15,27]. The combination of induced Activin-responsive genes and reduced myogenic regulators is most consistent with the suppression of early myogenic commitment rather than direct evidence of complete regenerative failure. SB431542 attenuated the Activin A-associated transcriptional changes, supporting the involvement of SB431542-sensitive signalling. However, because this inhibitor targets ALK4, ALK5, and ALK7, these findings do not identify the specific receptor mediating the observed response [28]. Receptor-specific genetic or selective pharmacological perturbation was not performed in the present study and would be required to establish receptor dependence.

Based on the integrated computational and experimental evidence, we propose a model in which ageing-associated macrophage state remodelling may alter the local regenerative niche through INHBA/Activin A-associated signalling (Figure 8). In this proposed framework, inflammatory macrophages represent a potential ligand source population, whereas epMuSCs represent a candidate recipient state implicated by the transcriptomic analyses. Whether epMuSCs respond more strongly to Activin A than other MuSC states remains to be determined. Rather than acting as a single determinant of muscle ageing, INHBA/Activin A signalling may represent one component of a broader immune–regenerative communication network that shapes regenerative state transitions during ageing.

Several limitations should be acknowledged. The transcriptomic datasets were cross-sectional and derived from independent cohorts, and NicheNet provides predictive rather than causal evidence. Although the THP-1-derived macrophage experiments provide evidence for *INHBA* expression and extracellular Activin A immunoreactivity, the maturation state and biological activity of the detected protein were not directly established. Experiments using primary MuSCs from three donors assessed responses to recombinant Activin A but did not reproduce the multicellular niche or establish a causal effect of macrophage-derived Activin A. Because Activin A responses were not compared among qMuSCs, epMuSCs, and differentiating MuSCs, the present functional experiments do not establish preferential responsiveness of epMuSCs. Direct testing will require functional conditioned medium experiments incorporating macrophage-specific *INHBA* perturbation or Activin A neutralization, together with the confirmation of pathway activity and regenerative outcomes in vivo.

Taken together, these findings suggest that regenerative decline in ageing muscle is not solely a consequence of MuSC-intrinsic dysfunction but may also arise from disrupted communication between immune cell states and regenerative state transitions. The combined computational, tissue, and primary cell evidence supports macrophage-associated INHBA/Activin A signalling as a candidate niche-modifying mechanism linking immune remodelling to altered MuSC state transitions within the ageing muscle niche.

## 4. Methods and Materials

### 4.1. Human Tissue Specimens and Primary Satellite Cell Isolation

This study was conducted in accordance with the Declaration of Helsinki and was approved by the institutional ethics committee. Written informed consent was obtained from all participants. Gracilis muscle tissue from a 65-year-old healthy male donor was used for tissue immunofluorescence staining. Primary satellite cells were isolated from skeletal muscle specimens obtained from three independent donors. Detailed clinical information of human donors is provided in Table S3.

Skeletal muscle specimens were minced and digested with DMEM containing 2 mg/mL dispase II (Roche, Basel, Switzerland) and 2 mg/mL collagenase type II (Sigma-Aldrich, St. Louis, MO, USA) for 90 min at 37 °C. The resulting cell suspensions were seeded and expanded to approximately 80–90% confluence before fluorescence-activated cell sorting. After staining for CD56 and CD82 and exclusion of debris and doublets, CD56^+^CD82^+^ human muscle satellite cells were isolated by fluorescence-activated cell sorting. The sorted cells were expanded, and cells at passages 3–4 were used for subsequent experiments.

### 4.2. Bulk RNA Sequencing Data and Analysis

A publicly available human skeletal muscle bulk RNA-seq dataset (GSE167186) was obtained from the Gene Expression Omnibus (GEO) database [29]. Samples annotated as young healthy (*n* = 19) or old healthy (*n* = 29) were retained for subsequent analyses. Differential expression analysis was performed using DESeq2, with old healthy versus young healthy defined as the comparison. Differentially expressed genes (DEGs) were identified using a nominal *p* value < 0.05 and an absolute log2 fold change ≥ 0.25. Variance-stabilizing transformation (VST) was applied for principal component analysis (PCA) and expression-based visualization.

Gene Ontology (GO) and Kyoto Encyclopedia of Genes and Genomes (KEGG) enrichment analyses were performed using clusterProfiler 4.6.2 [30]. Gene set enrichment analysis (GSEA) was conducted using hallmark gene sets from the Molecular Signatures Database (MSigDB), with genes ranked according to the DESeq2 Wald statistic [31]. Pathway activity at the individual sample level was further assessed using gene set variation analysis (GSVA), and differences between the young and old groups were evaluated using limma 3.54.2 [32].

Immune cell infiltration was estimated using CIBERSORT v1.03 with the LM22 signature matrix [33]. The analysis was performed with 1000 permutations and quantile normalization disabled (QN = FALSE). Estimated immune cell proportions were subsequently compared between the young healthy and old healthy groups.

### 4.3. Single-Cell RNA Sequencing Data and Analysis

Publicly available human skeletal muscle single-cell RNA-sequencing data were obtained from the single-cell component of the Human Skeletal Muscle Aging Atlas dataset [2], whereas snRNA-sequencing data were excluded. A total of 90,902 cells were extracted, including 41,261 cells from young donors (*n* = 5) and 49,641 cells from old donors (*n* = 7). Donors aged 20–40 years were classified as young, whereas those aged 60–75 years were classified as old. Cells with 300–5000 detected genes, 500–30,000 RNA counts, and ≤10% mitochondrial transcripts were retained, and cells annotated as doublets were excluded.

Following quality control, gene expression data were normalized using LogNormalize, and 3000 highly variable genes were identified. The data were scaled with total RNA counts and mitochondrial transcript percentages regressed out, followed by principal component analysis. Batch effects across samples were corrected using Harmony [34]. The first 30 Harmony dimensions were used for nearest-neighbour graph construction, UMAP visualization, and graph-based clustering at a resolution of 0.8. Cell identities were manually assigned according to canonical marker genes and reference annotations from the original dataset.

MuSCs and myeloid cells were subsequently extracted and independently re-clustered. MuSCs were classified into quiescent MuSCs (qMuSCs), epMuSCs, and differentiating MuSCs (dMuSCs). Myeloid cells were further annotated as inflammatory macrophages, lipid associated macrophages, resident macrophages, monocytes, neutrophils, dendritic cells, and cycling macrophages according to canonical marker expression.

Senescence-associated scores were calculated at the single-cell level using the SenMayo gene signature and the Seurat AddModuleScore function. Analyses were restricted to cells from old donors. For each donor and cell state, the median SenMayo score was calculated, and donor–state combinations represented by fewer than 10 cells were excluded. Differences among cell states were subsequently assessed using linear mixed-effects models, with cell state as a fixed effect and donor identity as a random effect.

### 4.4. Donor-Level Pseudobulk Differential Expression Analysis

To account for biological replication at the donor level, pseudobulk differential expression analysis was performed for epMuSCs. Donors represented by fewer than 20 cells within a given population were excluded. Raw transcript counts from cells belonging to the same donor and cell population were summed to generate donor-level pseudobulk count matrices.

Differential expression between old and young donors was analysed using edgeR [35]. Lowly expressed genes were removed using filterByExpr, followed by normalization and quasi-likelihood generalized linear modelling. DEGs were defined using a nominal *p* value < 0.05 and an absolute log2 fold change ≥ 0.25. The complete epMuSC pseudobulk DEG dataset is provided in Table S5. Functional enrichment analyses, including GO, KEGG, and hallmark GSEA, were subsequently performed using clusterProfiler and MSigDB gene sets [30,31].

### 4.5. NicheNet Ligand–Target Analysis

NicheNet was used to predict signalling from inflammatory macrophages, defined as sender cells, to epMuSCs, defined as receiver cells [36,37]. All sender and receiver cell expression analyses were restricted to cells from old donors. Genes detected in at least 5% of the corresponding cell population were considered expressed. The receiver gene set comprised genes upregulated in old relative to young epMuSCs in the donor-level pseudobulk analysis, whereas genes expressed in old epMuSCs served as the background gene set.

Candidate ligands were required to be expressed in sender cells, have corresponding receptors expressed in receiver cells, be represented in the NicheNet ligand–receptor network, and be classified as secreted factors. Ligand activity was quantified using Pearson correlation between predicted ligand regulatory potential and the ageing-associated epMuSC gene set. Top-ranked signalling axes were evaluated by comparing ligand expression across macrophage subtypes and receptor expression across MuSC states. The complete activity ranking of secreted candidate ligands is provided in Table S6. For INHBA, candidate receptors were further evaluated according to their average expression and the proportion of receptor-positive cells in old epMuSCs. Predicted INHBA targets were examined according to their regulatory potential and old-versus-young log2 fold change in epMuSCs.

### 4.6. Independent Older Adult Cohort Assessment of the INHBA-Associated Macrophage–epMuSC Signalling Framework

An independent human skeletal muscle single-cell RNA-sequencing dataset (GSE143704) was analysed to obtain older adult cohort support for the NicheNet-predicted INHBA-associated macrophage–epMuSC signalling framework identified in the discovery cohort. Donor age information was obtained from the original dataset metadata. To assess whether the identified cellular expression framework was present in an independent older adult human muscle cohort, only samples from donors aged ≥ 50 years were included. The final dataset comprised 18,320 cells from nine donors aged 54–81 years.

The expression matrix and metadata were obtained from the Gene Expression Omnibus (GEO) database and processed using Seurat 4.3.0. Macrophage and MuSC populations were extracted according to cell-type annotations and independently analysed. Inflammatory macrophages were retained as the population of interest, whereas other macrophage populations were grouped as “Other” for focused assessment of INHBA expression. MuSC populations were analysed to identify epMuSC states and evaluate expression of Activin receptor components, including ACVR1B, ACVR2A, and ACVR2B.

NicheNet analysis was performed using the same ligand–target framework as described above, with inflammatory macrophages as sender cells and epMuSCs as receiver cells. The ageing-associated epMuSC target programme derived from the discovery cohort was used as the receiver gene set, whereas genes expressed in older adult cohort epMuSCs were used as the background gene set. The analysis was performed to evaluate support for the INHBA-associated macrophage–epMuSC signalling framework in an independent older adult human muscle cohort.

### 4.7. THP-1-Derived Macrophage Polarization and INHBA/Activin A Analysis

THP-1 cells (MeisenCTCC, Hangzhou, China) were cultured in RPMI 1640 medium containing 10% foetal bovine serum. Cells were seeded in six-well plates at a density of 1.5 × 10^6^ cells per well and differentiated with 50 ng/mL PMA for 24 h, followed by a 24 h rest in fresh medium. M1-like polarization was induced with 100 ng/mL LPS and 20 ng/mL IFN-γ for 24 h, whereas M0-like macrophages were maintained without additional polarizing stimuli. Following polarization, cells were collected for RT-qPCR analysis of INHBA expression, and the corresponding culture supernatants were collected for Activin A measurement. Activin A concentrations were measured using a Human Activin A QuickTest ELISA Kit (FineTest, Wuhan, China; catalogue No. QT-EH0028) according to the manufacturer’s instructions. ELISA samples were assayed in duplicate in three independent macrophage differentiation and polarization experiments.

### 4.8. Activin A Treatment and Pathway Inhibition

Primary human MuSCs were assigned to three experimental groups: control, Activin A, and Activin A + SB431542. Recombinant human Activin A (MedChemExpress, Monmouth Junction, NJ, USA) was administered at a final concentration of 100 ng/mL. SB431542 (MedChemExpress, USA), an inhibitor of ALK4/5/7 signalling, was used at a final concentration of 1 μM.

For the combined treatment group, cells were pretreated with SB431542 for 1 h before Activin A stimulation. Cells were subsequently cultured in the continued presence of the indicated treatments for 48 h. Control cells received an equivalent volume of vehicle-containing culture medium. At the end of the 48 h treatment period, cells were either collected for RT-qPCR analysis or fixed for MYOG immunofluorescence staining.

### 4.9. Tissue Immunofluorescence Staining

Tissue immunofluorescence staining was performed to validate the presence and cellular localization of INHBA-positive macrophages in aged human skeletal muscle tissue. Muscle samples were fixed in 4% paraformaldehyde, paraffin-embedded, and sectioned at a thickness of 4 μm. Following deparaffinization and rehydration, heat-induced antigen retrieval was performed using Tris–EDTA buffer (Beyotime, Shanghai, China). Sections were then incubated with an immunofluorescence permeabilization and blocking buffer (Beyotime, Shanghai, China).

Sections were incubated with primary antibodies overnight at 4 °C, followed by incubation with species-matched fluorophore-conjugated secondary antibodies and DAPI for 1 h at room temperature in the dark. Fluorescence images were acquired using an Olympus fluorescence microscope (Olympus Corporation, Tokyo, Japan) under identical acquisition settings. Co-localization of macrophage markers and INHBA was assessed based on overlapping fluorescence signals. Detailed information regarding antibodies is provided in Table S1.

### 4.10. RNA Extraction, Reverse Transcription, and RT-qPCR

Total RNA was extracted from treated primary human MuSCs and THP-1-derived M0- and M1-like macrophages using the Super FastPure Cell RNA Isolation Kit (Vazyme Biotech Co., Ltd., Nanjing, China) according to the manufacturer’s instructions. RNA concentration and purity were determined using a spectrophotometer before reverse transcription. Complementary DNA (cDNA) was synthesized using HiScript IV All-in-One Ultra RT SuperMix for qPCR (Vazyme Biotech Co., Ltd., China). Quantitative real-time PCR was performed using ChamQ Universal SYBR qPCR Master Mix (Vazyme Biotech Co., Ltd., China) on an Applied Biosystems QuantStudio 5 Real-Time PCR System (Thermo Fisher Scientific, Waltham, MA, USA).

The amplification program consisted of an initial denaturation at 95 °C for 30 s, followed by 40 cycles of amplification at 95 °C for 10 s and 60 °C for 30 s with fluorescence acquisition. A melting curve analysis was subsequently performed to verify amplification specificity. Expression levels of Activin A-responsive and MuSC-associated genes in primary human MuSCs, as well as INHBA expression in THP-1-derived macrophages, were quantified, normalized to ACTB, and calculated using the 2^−ΔΔCt^ method. Primary MuSC experiments were performed using cells derived independently from three donors, whereas macrophage RT-qPCR experiments were performed in three independent differentiation and polarization experiments. Primer sequences are provided in Table S2.

### 4.11. Immunofluorescence Staining of Primary Human MuSCs

Primary human MuSCs after indicated treatments were fixed with 4% paraformaldehyde, permeabilized and blocked with an immunofluorescence permeabilization and blocking buffer (Beyotime, China). Cells were incubated with anti-MYOG antibody overnight at 4 °C, followed by fluorophore-conjugated secondary antibodies and DAPI staining. MYOG fluorescence intensity was quantified using ImageJ 1.54p. One field was randomly selected from each sample for quantification. Experiments were performed using MuSCs derived from three independent donors.

### 4.12. Statistical Analysis

Statistical analyses were performed using R version 4.2.2. All tests were two-sided, and *p* < 0.05 was considered statistically significant. The donor was treated as the unit of biological replication in single-cell and primary-cell analyses. For single-cell-derived donor-level analyses, differences among cell states were evaluated using linear mixed-effects models with cell state as a fixed effect and donor identity as a random effect. Pairwise comparisons were performed using estimated marginal means with Benjamini–Hochberg correction. *p* values from GO, KEGG, GSEA, and GSVA analyses were adjusted using the Benjamini–Hochberg method. *p* values from CIBERSORT-derived immune cell fractions and donor-level cell proportion comparisons were reported.

For RT–qPCR analysis, technical replicate Ct values were averaged before ΔCt values were calculated as Ct_target_ − Ct_ACTB_. The donor was treated as the independent biological unit, and all comparisons were donor matched. For SERPINE1, BHLHE40, MYOD1, and MYOG, inferential testing was restricted to two biologically motivated contrasts: Activin A versus vehicle control and Activin A plus SB431542 versus Activin A. Two-sided paired *t*-tests were performed on ΔCt values. The resulting eight *p* values, comprising four genes and two contrasts per gene, were adjusted collectively using the Benjamini–Hochberg procedure to control the false discovery rate. Benjamini–Hochberg-adjusted *p* values < 0.05 were considered statistically significant. Relative expression was calculated using the 2^−ΔΔCt^ method, with the donor-matched control condition used as the calibrator. Data are presented as the mean ± SEM from three independent donors, and individual data points represent donor-level biological replicates.

For macrophage RT-qPCR analysis, paired M0- and M1-like conditions generated within the same independent experiment were compared using two-sided paired *t*-tests. M0-like macrophages were used as the calibrator for relative INHBA expression. ELISA results are presented descriptively as blank-corrected OD450 values (mean ± SD). Samples with signals below the lowest non-zero standard were not converted to numerical concentrations.

For MYOG immunofluorescence analysis, donor-matched paired comparisons were performed between the indicated treatment groups, with *p* values adjusted using the Benjamini–Hochberg method for multiple comparisons.

## Data Availability

The bulk RNA-sequencing data analysed in this study are publicly available from the Gene Expression Omnibus (GEO) database under accession number GSE167186. The scRNA-seq data were obtained from the Human Skeletal Muscle Aging Atlas and are publicly available through the original study. The independent older adult cohort dataset was obtained from GEO accession GSE143704.

## Supplementary Materials

The following supporting information can be downloaded at: https://www.mdpi.com/article/10.3390/ijms27188283/s1.
